# Perceived Stress and Disordered Eating Behaviors in Emerging Adulthood: A Multidimensional Pilot Study of Dietary and Genetic Factors

**DOI:** 10.3390/diseases14090314

**Published:** 2026-08-28

**Authors:** Evgeniya Klein, Daria Velina, Irina Stanislavovna Kolesnikova, Valeriy Vladimirovich Polunovskiy, Nina Vitalievna Panteleeva, Dmitry Alexandrovich Kulikov, Alla Nikolaevna Stolyarova, Igor Nikitin

**Affiliations:** 1 Department of Food Technology and Bioengineering, Plekhanov Russian University of Economics, 36 Stremyanny per., 115054 Moscow, Russia; kattim67@gmail.com; 2 National Center for Genetic Research, Nikolayeva St., 12, Floor, Unit 6, 18, 630090 Novosibirsk, Russia; i.kolesnikova@mygenetics.ru (I.S.K.); v.polunovskiy@mygenetics.ru (V.V.P.); n.panteleeva@mygenetics.ru (N.V.P.); 3 Rogov Institute of Applied Biotechnology and Food Engineering, Federal State Budgetary Educational Institution of Higher Education Russian Biotechnological University, 11 Volokolamsk Highway, 125080 Moscow, Russia; kulikovda@mgupp.ru; 4 Basic Department of Trade Policy, Plekhanov Russian University of Economics, 36 Stremyanny per., 115054 Moscow, Russia; stolyarova.an@rea.ru; 5 Department of Biotechnology of Food Products from Plant and Animal Raw Materials, K.G. Razumovsky Moscow State University of Technologies and Management (The First Cossack University), Zemlyanoy Val, 73, 109004 Moscow, Russia

**Keywords:** added sugar, disordered eating behaviors, emotional eating, gene–environment interaction, perceived stress, young women

## Abstract

Background: Subclinical disordered eating behaviors (DEBs) are common among young women and are thought to result from complex interactions between psychological, dietary, and genetic factors. While chronic stress and unhealthy dietary habits have been implicated in the development of DEBs, the independent and interactive contributions of perceived stress, added sugar intake, and genetic susceptibility remain insufficiently understood. This pilot study investigated these associations using a two-stage case–control design. Methods: A total of 100 female university students (18–27 years) completed the Dutch Eating Behavior Questionnaire (DEBQ) during the first stage of the study. Based on DEBQ scores, 20 participants with the highest risk of DEBs and 20 with the lowest risk were selected for the second stage, forming case and control groups. Perceived stress was assessed using the Perceived Stress Scale (PSS-14), and added sugar intake was estimated using a semi-quantitative food frequency questionnaire (FFQ). Participants were also genotyped for four candidate polymorphisms (5-HTTLPR, rs6295, rs6265, and rs1800497). Group differences, correlation analyses, binary logistic regression models adjusted for BMI, and gene–environment interaction analyses were performed. Results: Perceived stress emerged as the strongest and most consistent predictor of belonging to the upper DEBQ quintile, with each one-point increase on the PSS-14 associated with a 44% increase in the odds of high-risk status after adjustment for BMI (OR = 1.44; 95% CI: 1.120–1.853; *p* = 0.004). Added sugar consumption did not withstand correction for multiple comparisons (*p* = 0.030, Bonferroni-adjusted α = 0.025). No significant main effects or gene–environment interactions were detected for any of the investigated polymorphisms or the cumulative genetic risk score. Conclusions: These preliminary findings highlight perceived stress as the important modifiable risk factor for subclinical disordered eating behaviors in young women, suggesting that stress-reduction and emotional regulation interventions may be more effective than dietary approaches in this population. However, the lack of significant associations for the genetic variants studied should be interpreted with caution given the limited sample size and statistical power. Replication in larger, longitudinal cohorts is warranted to confirm these findings and explore developmental trajectories.

## 1. Introduction

Disordered eating behaviors (DEBs) represent a complex and multifaceted issue in contemporary public health that extends far beyond individual psychological difficulties and affects people of widely varying ages, social statuses, ethnicities, and educational levels [1]. In the scientific literature and clinical practice, traditional attention has been predominantly directed toward severe psychiatric diagnoses, such as anorexia nervosa, characterized by deliberate dietary restriction and emaciation, and bulimia nervosa, manifesting in recurrent episodes of binge eating followed by compensatory behaviors—these conditions have been thoroughly investigated with respect to etiology [2], pathogenesis [3], and therapeutic approaches [4]. However, despite the full gravity of clinically delineated disorders, so-called subclinical forms of DEBs are considerably more prevalent in the general population; these do not fit within the rigid boundaries of formal diagnoses yet exert an equally detrimental impact on physical and mental health over the long term [5]. Such subclinical manifestations include, in particular, restrictive eating, understood as chronic, often obsessive caloric restriction and persistent self-monitoring of food intake [6]; emotional eating, representing a maladaptive coping mechanism for stress, anxiety, boredom, or sadness, wherein eating becomes a means of regulating affective states rather than satisfying a physiological need for energy [7]; and external eating, driven by heightened sensitivity to external food cues–smell, sight, and availability of food–which become triggers for food consumption even in the absence of hunger [8]. It is precisely these socially acceptable patterns of eating behavior that, due to their widespread prevalence and chronic course, are increasingly recognized as risk factors that create fertile ground for the subsequent development of full-fledged psychopathology and for the onset of obesity, type 2 diabetes mellitus, and other metabolic disorders [9,10].

Early identification and correction of subclinical forms of DEBs thus become critical for preventing the development of severe diseases. In this regard, attention should be paid to the potential predisposing factors underlying the emergence of eating-related behavioral disturbances.

First, as in the case of clinical eating disorders (EDs), biological factors, in particular genetic predisposition, may contribute significantly to the etiology of DEBs. The literature has highlighted a number of candidate genes whose polymorphisms are associated with an increased risk of anorexia nervosa, bulimia nervosa, and binge eating disorder [11]. For instance, the polymorphic 5-HTTLPR region of the serotonin transporter gene (5-HTT, *SLC6A4*) has been associated with an elevated risk of restrictive eating behavior and anorexia nervosa: the short (S) allele reduces the efficiency of gene transcription, thereby impairing neurotransmission of the 5-HTT transporter protein, which is functionally linked to the control of hunger signals and anxiety [12]. Alongside this, the literature indicates that the presence of the S-allele is associated with heightened stress reactivity and impulsivity, which is in turn linked to an increased risk of developing emotional eating [13].

Also worthy of attention is the functional C(−1019)G polymorphism (rs6295) of the *HTR1A* gene, which encodes the presynaptic 5-HT1A autoreceptor. This receptor serves as a key regulator of serotonergic activity, mediating a negative feedback mechanism: the higher its functionality, the stronger the inhibition of serotonin release into the synaptic cleft [14]. The G-allele modulates the expression of this autoreceptor, which appears to alter feedback sensitivity and leads to an imbalance in serotonergic neurotransmission. Clinically, this manifests as increased anxiety and rigidity (obsessive-compulsive traits), which, in turn, constitute the neuropsychological basis of vulnerability to anorexia nervosa and other EDs [15].

In the context of the genetics of eating behavior, the rs6265 (Val66Met) polymorphism of the *BDNF* gene, which encodes brain-derived neurotrophic factor, is frequently examined. This protein serves as a key factor in neuroplasticity and is also involved in the integration of energy metabolism signals and the regulation of eating behavior [16]. The substitution of valine by methionine attenuates activity-dependent release of *BDNF* at synapses, thereby impairing inhibitory control over appetite, giving rise to emotional instability, and distorting the valuation of food-related stimuli [17]. For instance, evidence indicates that female carriers of the Met-allele with diagnosed anorexia nervosa assign a more positive appraisal to fasting [18], whereas patients who have undergone bariatric surgery and carry the Met-allele are more prone to binge eating [19].

Another important genetic factor is the rs1800497 (Taq1A) polymorphism, located in the *ANKK1* gene and closely associated with the functioning of dopamine D2 receptors (*DRD2*). Carriage of the A1 allele (G → A, C → T) has been linked to reduced D2 receptor density in the striatum [20], which may result in diminished sensitivity of the reward system and altered food-related motivation. This increases the likelihood of developing maladaptive eating patterns, including hedonic eating, emotional overeating, and food addiction [21,22]. Evidence indicates that the A1 allele is associated both with higher body mass and with excessive consumption of sugar and calories, with these phenotypic manifestations appearing to be interrelated [23].

The presence of genetic risk variants alone is not a sufficient condition for the development of DEBs or Eds—their negative contribution materializes only through interaction with adverse environmental factors, among which stress plays a leading role. Chronic exposure to stressors triggers a cascade of neuroendocrine reactions mediated by elevated cortisol secretion and dysregulation of the hypothalamic–pituitary–adrenal (HPA) axis, which in turn affects dopaminergic system activity [24], the balance of appetite-regulating hormones and the efficacy of satiety signaling [25], as well as gastrointestinal motility and glucose sensitivity [26]. At the behavioral level, this may manifest either as reduced appetite under acute stress or as overeating under chronic stress, wherein food is used as an accessible means of alleviating anxiety and suppressing negative emotions [27].

Stress-induced overeating sustains and exacerbates DEBs, as many of the traditional “comfort” foods consumed to dampen negative emotions are sources of saturated fats and sugars [28], which exert a range of adverse effects on the organism: first, they alter the composition of the gut microbiota, suppressing beneficial microorganisms and promoting the growth of pro-inflammatory strains (inflammation being a risk factor for depression and other mental disorders [29,30]; second, these dietary components produce a brief, powerful dopamine surge that over time blunts the sensitivity of the reward system, compelling the individual to consume increasingly larger amounts to achieve the same effect, thereby fostering food addiction—a condition clinically manifested by loss of control over the quantity of food consumed [31,32]. Importantly, excessive consumption of saturated fats and sugars does not always correlate with elevated stress—it may also result from poorly established eating habits and/or a forced lifestyle in the context of university studies or intensive work activity [33].

The present observational study is aimed at integrating the aforementioned biological and environmental factors to assess their individual and combined contribution to the development of DEBs. Given the objectives set, it was decided to conduct the study in two stages: the first stage involves the analysis of a cohort of 100 female participants, from which 20 participants with the highest risk of DEBs (cases) and 20 participants with the lowest risk of DEBs (controls) are selected. The study hypothesis posits that participants in the case group exhibit significantly higher levels of subjectively perceived stress and consumption of foods high in added sugar compared to the control group, with the genetic component modifying this effect. The choice of sugar-containing products as a marker of “comfort food” is justified by the more extensively studied and pronounced effect of sugar on the dopaminergic reward system [34] relative to other taste components, particularly fats, salt, and monosodium glutamate. The selection of genetic variants is hypothesis-driven and focused on signaling pathway analysis. Specifically, polymorphisms are selected to represent serotonergic signaling (5-HTTLPR and *HTR1A* rs6295), neurotrophic regulation (*BDNF* rs6265), and dopaminergic signaling (*ANKK1/DRD2* rs1800497). Accordingly, the present study aimed to examine individual biological pathways potentially relevant to DEBs rather than to comprehensively assess the genetic contribution to these behavioral responses.

The presented study is a pilot in nature and is intended to identify key patterns for subsequent in-depth verification on an expanded sample.

## 2. Materials and Methods

### 2.1. Study Participants

The study consisted of two stages: in the first stage, 100 participants were recruited for a cross-sectional study; in the second stage, 40 individuals were selected from the previously recruited participants according to the criteria outlined below for allocation into case and control groups.

#### 2.1.1. Participants in the Cross-Sectional Study

The study participants were 100 women aged 18–27 years. All participants were recruited from among university students in Moscow, with invitations to participate distributed via social media and messaging applications. The main inclusion criteria were female sex, age 18–27 years, residence in Moscow, and willingness to provide genetic material if selected for the second stage of the study. Women meeting the following exclusion criteria were not included: use of appetite-suppressing medications; presence of acute clinical conditions associated with EDs. Participants were not excluded on the basis of BMI (except in cases where critical underweight was observed in the context of diagnosed anorexia nervosa), as subclinical DEB identified via the Dutch Eating Behavior Questionnaire (DEBQ) is not confined to a specific phenotype and may be observed both in women with moderately reduced body weight and in those with overweight.

#### 2.1.2. Participants in the Case–Control Study

In the framework of this study, 20 participants who completed the DEBQ during the first stage and met the following criteria were classified as “cases”:

- the total DEBQ score falls within the upper quintile of the sample distribution;
- the scores on the emotional eating subscale are not below the median value.

Participants with a high total DEBQ score but low scores on the emotional eating subscale were excluded from the study, as the study was focused on stress-induced disordered eating, which are primarily captured by this subscale.

The control group comprised 20 participants with the lowest overall risk of disordered eating (lower quintile on the DEBQ), whose emotional eating scores did not exceed the median threshold.

Thus, in the case–control component, an extreme-groups sampling strategy was employed, based on the upper and lower quintiles of the DEBQ distribution. This approach was chosen to enhance phenotypic contrast and statistical efficiency within this pilot study. As participants were not selected according to population-based case–control sampling procedures, the effect estimates were not interpreted as population-level risk estimates.

### 2.2. Study Procedures

All procedures were approved by the Local Ethics Committee of K.G. Razumovsky Moscow State University of Technologies and Management (Protocol No. 6 dated 5 May 2026).

Participants in the first stage of the study were asked to complete the DEBQ electronic questionnaire; consent for the transfer and further processing of the questionnaire results was given electronically by checking the appropriate box before clicking the “submit form” button.

Participants in the second stage of the study were asked to complete two electronic questionnaires—the Perceived Stress Scale (PSS-14) and a Food Frequency Questionnaire (FFQ)—as well as to provide a genetic test for the identification of risk alleles of the rs6295, rs6265, rs1800497, and 5-HTTLPR polymorphisms. Prior to the commencement of this stage of the study, written informed consent was obtained from each participant.

To ensure participant comfort, participants were asked to independently collect epithelial cells from the buccal surface using sterile tampon probes; the procedure was performed under the supervision of a member of the research team in a designated room at the university. The collected biological material was then dried and transferred for analysis to a genetic laboratory, with each sample assigned a number in the NN00XX format and a unique five-digit code, thereby precluding access to biometric data of all individuals except the principal investigator.

### 2.3. Study Measures

#### 2.3.1. Disordered Eating Behaviors

Participants’ eating behavior was assessed using the Dutch Eating Behavior Questionnaire (DEBQ), which comprises three subscales: restrained, emotional, and external eating [35]. The “Restrained Eating” subscale evaluates the degree of conscious control over food intake aimed at body weight management; high scores on this subscale indicate a tendency toward dieting, intentional caloric restriction, and suppression of the urge to eat. The “Emotional Eating” subscale measures the propensity to overeat in response to emotional states such as anxiety, stress, depression, boredom, or loneliness; high scores on this subscale point to a tendency to use food as a coping strategy for negative emotions. The “External Eating” subscale reflects sensitivity to external food cues (sight, smell, food availability) and the tendency to initiate eating in response to external signals while disregarding internal feelings of hunger and satiety; high scores on this subscale characterize reactivity to environmental food stimuli. Data processing was carried out in accordance with the standard procedure. Each questionnaire item was rated by respondents on a 5-point Likert scale (ranging from 1—“never” to 5—“very often”). Scoring was performed in several stages:

- For each subscale separately, a raw total score was computed by summing all responses to the items comprising that subscale.
- To correct for differences in the number of items across subscales, a mean score was additionally calculated for each subscale (the sum of scores on the subscale divided by the number of items in that subscale), which allowed for a valid comparison of the severity of different types of eating behavior with one another.

In addition to the scores on individual subscales, an overall total score for the entire questionnaire was computed by summing the mean scores of all three subscales to provide an integrated assessment of the general propensity toward eating disturbances. The higher the overall total score, the more unfavorable the participant’s eating behavior was considered. This approach has been used in previous studies [36,37], where a higher total score served as a screening tool to identify individuals with the most problematic eating behavior.

#### 2.3.2. Subjectively Perceived Stress Level

The level of subjectively perceived stress was assessed using the Perceived Stress Scale (PSS-14) [38]. This instrument is one of the most widely used in global psychology for measuring the extent to which various life situations are appraised by an individual as stressful, unpredictable, overwhelming, or uncontrollable [39].

The questionnaire consists of 14 items, each reflecting the frequency of experiencing specific thoughts and feelings related to stressful states over the past month. The items are designed to assess two main aspects of perceived stress:

- The negative affectivity/distress subscale measures the degree of experiencing negative emotional states, feelings of loss of control over one’s life, accumulated irritability, and emotional exhaustion.
- The positive affectivity/coping subscale reflects the ability to feel self-confidence, a sense of control over events, and the capacity to cope with emerging difficulties.

Data processing was conducted in accordance with the standard procedure recommended by the authors of the instrument. Each statement was rated by respondents on a 5-point Likert scale, with responses ranging from 0—“never” to 4—“very often.” Importantly, the questionnaire contains both positively and negatively worded items; therefore, scoring was performed in several stages:

- Reverse coding of responses to positively framed items (items 4, 5, 6, 7, 9, 10, and 13 according to the standard scoring key): scores for these items were reversed (0 ↔ 4, 1 ↔ 3, 2 ↔ 2) so that all items were consistently oriented toward higher levels of perceived stress.
- Calculation of the total score for the entire questionnaire by summing all 14 recoded responses. The possible range of scores was from 0 to 56 points.

Interpretation of the results was as follows: the higher the final total score, the higher the level of subjectively perceived stress.

#### 2.3.3. Added Sugar Consumption

The assessment of added sugar consumption was conducted using a specially designed semi-quantitative Food Frequency Questionnaire (FFQ) developed for the Russian population. The questionnaire comprised 15 items covering the main categories of foods that serve as sources of added sugar in the typical Russian diet: confectionery products (chocolate, candies, cookies, cakes, pastries), sugar-sweetened beverages (fruit juices, carbonated soft drinks), industrially produced cereals and breakfast items (instant cereals, flakes, muesli), as well as dairy and fermented dairy products with added sugars (milkshakes, sweetened yogurts, and curd snacks).

For each food item, respondents were asked to indicate the frequency of consumption over the preceding three months using a standard portion size specified for each product. Portion sizes were selected to facilitate easy estimation by participants and included commonly recognizable units, such as pieces of chocolate, individual candies and cookies, slices of cake, individual pastries, and glasses of juice or carbonated beverages. Standard portion sizes were based on typical consumption patterns in the Russian population.

To ensure accurate quantification of added sugar intake, only added sugars were assessed; while naturally occurring sugars (e.g., those present in dried fruits or other unsweetened products) were not included in the calculations. For products with added sugar, the sugar content per standard portion was determined using manufacturer’s nutritional information provided on product packaging and, where necessary, supplemented with data from national food composition tables for the Russian Federation. For sugar independently added by respondents to hot beverages (tea, coffee) and other dishes, the amount was assessed as the number of teaspoons (with one teaspoon assumed to contain 5 g of sugar) and converted to grams based on the reported frequency of addition.

Data processing was carried out in stages. For each product, the frequency of consumption was converted into the average daily number of servings using the following coefficients:

“Never or less than once a month”—0;
“1–3 times a month”—0.067;
“Once a week”—0.143;
“2–4 times a week”—0.429;
“5–6 times a week”—0.786;
“Once a day”—1.0;
“2 or more times a day”—2.0;

Next, the resulting average daily number of servings was multiplied by the added sugar content in one standard serving of the corresponding product (in grams). Data on sugar content were obtained based on information provided by manufacturers on product packaging, as well as using reference tables on the chemical composition of food products. For sugar added independently to beverages and dishes, the amount was converted from the volume of a teaspoon (assumed to be 5 g of sugar) into grams, taking into account the average daily frequency of addition.

Total daily added sugar consumption for each respondent was calculated as the sum of the values for all products and the amount of sugar independently added. The resulting values were expressed in grams per day.

The FFQ was specifically designed for this study and was not formally validated against objective biomarkers of sugar intake. However, the questionnaire followed established principles for semi-quantitative FFQ design, including the use of standard portion sizes, frequency response categories, and a focus on a limited number of food items that represent the major sources of added sugar in the target population.

#### 2.3.4. Genotyping

The four polymorphisms were selected a priori based on previously reported associations with eating behavior, stress reactivity, appetite regulation, and reward-related processes. The candidate-gene panel was intended to represent selected serotonergic, neurotrophic, and dopaminergic pathways and was not intended to capture the overall genetic architecture of DEBs.

Buccal epithelium was collected by the participants themselves using special sampling kits (sterile tampon probes); after that, dried sealed materials were delivered to a laboratory by courier.

DNA was extracted by adsorption on silicon dioxide crystals (based on Boom et al., 1990 [40]). Genotyping of rs6295 (*HTR1A* gene), rs6265 (*BDNF* gene), rs1800497 (*DRD2* gene), rs11867581 and rs2129785 (5-HTTLPR gene promoter L/S polymorphism marker variants) were accomplished by PCR with hybridization-fluorescent real-time detection. PCR reactions were carried out using a CFX96 thermocycler (Bio-Rad Laboratories, Inc., Hercules, CA, USA) and Biolabmix PCR Mastermix (Biolabmix LLC, Novosibirsk, Russia). Primers, probes and amplification conditions were developed by National Center for Genetic Research LLC (Novosibirsk, Russia) and allowed for use for medical purposes as a part of the Metabolic Kit 60 (registration certificate No. P012-00110-77/00651190 dated 15 May 2023).

#### 2.3.5. Sample Size Determination

The sample size of 40 participants (20 cases and 20 controls) was selected based on the practical constraints of conducting a pilot study that involved multifaceted data collection, including genetic testing. As the primary aim was to identify potential trends and generate hypotheses rather than to provide definitive population estimates, a formal power calculation was not performed. The pilot design was considered appropriate for examining the feasibility of the study protocol and for estimating preliminary effect sizes, which can subsequently be used to design an adequately powered study with a larger cohort.

### 2.4. Statistical Analyses

Statistical analysis of the data was performed using the open-source software jamovi (version 2.6.44).

Given that quantitative variables in the overall sample (age, BMI, total DEBQ score) demonstrated a statistically significant deviation from normal distribution, as assessed by the Shapiro–Wilk test (*p* < 0.05), a non-parametric approach was adopted for all subsequent analyses. Descriptive statistics for continuous variables are presented as medians and interquartile ranges (Me [Q1; Q3]), while categorical data are expressed as frequencies and percentages (*n* (%)).

Comparisons between groups in the case–control sample were conducted using the Mann–Whitney U test. Effect sizes for the Mann–Whitney U test were quantified using the rank-biserial correlation coefficient (r).

To control for Type I error, Bonferroni correction was applied separately within pre-specified hypothesis families. For the two primary environmental comparisons (perceived stress and added sugar consumption), k = 2 and the adjusted significance threshold was α = 0.025. For the four individual candidate polymorphisms, k = 4 and the adjusted significance threshold was α = 0.0125. The two gene–environment interaction analyses (stress × cumulative genetic risk and added sugar × cumulative genetic risk) were considered as a separate hypothesis family, for which k = 2 and α = 0.025. The value of k therefore corresponded to the number of hypotheses within each predefined analysis family rather than to the total number of statistical tests performed in the study. Other analyses, including correlations and analyses of the cumulative genetic risk score as a secondary exploratory measure, were considered exploratory and are interpreted accordingly.

The association between genotype distribution and group membership was assessed using Fisher’s exact test due to small expected frequencies in some genotype categories. Hardy–Weinberg equilibrium (HWE) was assessed in the control group using the chi-square goodness-of-fit test (df = 1) and an exact test because of the small sample size and the presence of low expected genotype counts. The exact test was used as the primary assessment of HWE, with the chi-square results reported for comparison.

To evaluate the strength and direction of linear relationships between the main continuous variables, Spearman’s rank correlation coefficient (ρ) was calculated for the entire second-stage sample (*n* = 40).

To identify independent predictors of classification as a “case” (i.e., high risk of DEBs), binary logistic regression models were constructed. The dependent variable was group status (cases vs. controls). For each predictor (perceived stress, added sugar consumption, genetic risk), separate unadjusted (univariate) models were run, followed by models adjusted for BMI. The inclusion of BMI as a covariate was not prespecified in the protocol but was motivated by the observed difference in BMI between the case and control groups, indicating that BMI could potentially confound the relationships under study. Model fit was assessed using Nagelkerke’s pseudo-R^2^ (R^2^_N_).

Genetic risk was calculated as the sum of risk alleles across the four polymorphisms (5-HTTLPR, rs6295, rs6265, and rs1800497) using an additive model. For the sensitivity analysis, the cumulative genetic risk score was recalculated excluding rs6295, as this polymorphism showed a significant deviation from Hardy–Weinberg equilibrium in the control group.

Potential multicollinearity between BMI and the predictors included in the regression models was assessed using variance inflation factors (VIFs) and tolerance values. A VIF value below 5 and a tolerance value above 0.20 were considered indicative of the absence of substantial multicollinearity.

To evaluate gene–environment interactions, two fully adjusted (with BMI as a covariate) binary logistic regressions were constructed: the first with the product term stress × genetic risk, and the second with the product term added sugar × genetic risk. The significance of the interaction terms and the change in R^2^_N_ were assessed as measures of their additional explanatory value.

## 3. Results

### 3.1. Description of the Study Sample

#### 3.1.1. The First Stage

All study participants (*n* = 100) were women enrolled in higher education institutions in Moscow. Normality of the distribution of quantitative variables was assessed using the Shapiro–Wilk test. For age (W = 0.888; *p* < 0.001), body mass index (W = 0.962; *p* = 0.006), and total DEBQ score (W = 0.969; *p* = 0.019), a statistically significant deviation from normal distribution was detected. Accordingly, quantitative data are presented as medians and interquartile ranges (Table 1).

#### 3.1.2. The Second Stage

When assessing the distribution of variables within the study groups using the Shapiro–Wilk test, it was found that the distribution of age and total DEBQ score did not conform to normality in at least one of the groups (*p* < 0.05). Accordingly, quantitative data in Table 2 are presented as medians and interquartile ranges (Me [Q1; Q3]), and between-group comparisons were performed using the Mann–Whitney U test.

Participants in the case group demonstrated substantially higher scores across all three subscales compared to the control group. The most pronounced difference was observed for emotional eating (cases: median 3.35 vs. controls: 1.30), followed by restrained eating (cases: median 3.55 vs. controls: 1.55) and external eating (cases: median 3.75 vs. controls: 2.40). Exploratory group comparisons using the Mann–Whitney U test confirmed significant differences for all three subscales (all *p* < 0.001), with the largest effect size observed for emotional eating (r = −1.00), followed by restrained eating (r = −0.927) and external eating (r = −0.87).

Participants in the case group were characterized by higher levels of perceived stress compared to the control group (U = 61.5; *p* < 0.001). Furthermore, statistically significant differences between the groups were found for added sugar consumption (U = 117.0; *p* = 0.024). The differences in perceived stress were characterized by a large effect size (r = 0.693), while the differences in added sugar consumption demonstrated a moderate effect size (r = 0.415). However, when interpreting these results, adjustment for multiple comparisons should be taken into account: after Bonferroni correction (α = 0.025), the differences in stress retained statistical significance (*p* < 0.001), whereas the differences in sugar consumption were at the boundary of the accepted significance level (*p* = 0.024), warranting cautious conclusions regarding this variable.

Also, despite the absence of initial selection criteria based on BMI, higher BMI values were observed in the group of participants with higher risk of eating disturbances (U = 112.5; *p* = 0.018), reflecting the association of emotional and external eating with overeating and necessitating further consideration of BMI as a covariate in regression analyses.

To assess linear relationships between key variables in the overall sample (*n* = 40), Spearman’s correlation analysis was performed. A statistically significant positive correlation was found between the total DEBQ score and perceived stress levels (ρ = 0.516; *p* < 0.001), which is consistent with the results of between-group comparisons and confirms the close association between stress and the risk of DEBs. Statistically significant, albeit weaker, positive correlations were found between DEBQ and added sugar consumption (ρ = 0.318; *p* = 0.045), as well as between DEBQ and BMI (ρ = 0.325; *p* = 0.041). The correlation between stress levels and added sugar consumption was moderate in magnitude (ρ = 0.481; *p* = 0.002), which may reflect the potential role of stress in shaping cravings for sugar-containing products. The remaining correlations between the examined variables did not reach statistical significance (*p* > 0.05).

Analysis of genotype distribution in the sample revealed that heterozygosity was most common for 5-HTTLPR (70%) and rs6295 (70%), whereas for the rs6265 and rs1800497 polymorphisms, homozygosity for the neutral allele predominated, observed in 62.5% and 77.5% of participants, respectively (Table 3).

No statistically significant differences in the distribution of genotypes for the 5-HTTLPR, *HTR1A*, *BDNF*, and *DRD2* polymorphisms were found between the case group and the control group (Table 4). For the four individual candidate polymorphism comparisons, the Bonferroni-adjusted significance threshold was α = 0.0125.

HWE was assessed in the control group using both the chi-square goodness-of-fit test and an exact test, given the small sample size and low expected genotype counts. The genotype distributions were consistent with HWE for 5-HTTLPR (χ^2^ = 2.17, *p* = 0.140; exact *p* = 0.355), *BDNF* rs6265 (χ^2^ = 0.90, *p* = 0.343; exact *p* = 1.000), and *DRD2* rs1800497 (χ^2^ = 0.93, *p* = 0.335; exact *p* = 0.354). In contrast, a significant deviation from HWE was observed for *HTR1A* rs6295 (χ^2^ = 7.20, *p* = 0.007; exact *p* = 0.015). This deviation was taken into account when interpreting the rs6295 findings, which were considered exploratory and interpreted with caution.

### 3.2. Analysis of Independent Associations with the Risk of Disordered Eating Behaviors

#### 3.2.1. Stress and Disordered Eating Behaviors

The effect of stress on the risk of being classified into the high-DEB group was assessed using binary logistic regression. In the univariate model, stress was significantly associated with increased odds of membership in the high-DEB group (OR = 1.26; 95% CI: 1.088–1.461; *p* = 0.002; R^2^_N_ = 0.459). After adjustment for BMI, the odds ratio for stress increased to 1.44 (95% CI: 1.120–1.853; *p* = 0.004; R^2^_N_ = 0.686), with BMI also demonstrating a significant independent association with the outcome (OR = 1.88; 95% CI: 1.178–2.998; *p* = 0.008).

#### 3.2.2. Added Sugar Consumption and Disordered Eating Behaviors

For the data on between-group added sugar consumption, both a univariate model and a model accounting for BMI were constructed. It was found that added sugar consumption (each additional gram per day) increased the odds of high-DEB group membership by 6.4% (OR = 1.064; 95% CI: 1.006–1.125; *p* = 0.03; R^2^_N_ = 0.247), with the effect slightly strengthening after adding BMI to the model (OR = 1.09; 95% CI: 1.01–1.172; *p* = 0.033; R^2^_N_ = 0.453), indicating an independent contribution of sugar that is not accounted for by body weight. The contribution of BMI itself in the constructed model was more pronounced: OR = 1.5; 95% CI: 1.09–2.06; *p* = 0.013.

#### 3.2.3. Genotype and Disordered Eating Behaviors

For the regression model, a cumulative genetic risk score was used, reflecting the total number of risk alleles across the 5-HTTLPR, rs6295, rs6265, and rs1800497 polymorphisms in participants. In the univariate logistic regression analysis, cumulative genetic risk did not demonstrate a statistically significant association with probability of high-DEB classification (OR = 1.269; 95% CI: 0.75–2.14; *p* = 0.369; R^2^_N_ = 0.027). After adjustment for BMI, the effect remained non-significant (OR = 1.18; 95% CI: 0.68–2.06; *p* = 0.555; R^2^_N_ = 0.205). A sensitivity analysis was performed excluding the *HTR1A* rs6295 polymorphism from the cumulative genetic risk score, given its deviation from HWE in the control group. In the unadjusted logistic regression model, the association between the revised genetic risk score and high-DEB classification remained non-significant (OR = 1.203; 95% CI: 0.66–2.19; *p* = 0.546; R^2^_N_ = 0.012). After adjustment for BMI, the effect remained negligible and non-significant (OR = 1.07; 95% CI: 0.56–2.05; *p* = 0.840; R^2^_N_ = 0.196). These findings confirm that the inclusion of the *HTR1A* variant did not materially influence the observed genetic associations, and the exclusion of this polymorphism does not alter the overall conclusions of the study.

#### 3.2.4. Assessment of Multicollinearity

For each primary predictor (perceived stress, added sugar consumption, and cumulative genetic risk), separate unadjusted logistic regression models were first fitted, followed by models adjusted for BMI. Multicollinearity diagnostics revealed VIF values of 1.33 for perceived stress, 1.33 for added sugar consumption, and 1.01 for cumulative genetic risk, with all values substantially below the threshold of 5. All tolerance values were above 0.75. Thus, no significant redundancy was detected between BMI and the other predictors in the model.

### 3.3. Gene–Environment Interaction

To assess the potential interaction between perceived stress levels and cumulative genetic risk with respect to the risk of being classified into the high-DEB group, a binary logistic regression model adjusted for body mass index was constructed. In the model, only BMI demonstrated a statistically significant association with belonging to the high-risk group for disordered eating (OR = 2.05; 95% CI: 1.16–3.65; *p* = 0.014). For each 1 kg/m^2^ increase in BMI, the probability of a participant being classified into the high-risk group increased approximately twofold.

No statistically significant association was found between cumulative genetic risk and the likelihood of being in the high-risk group (OR = 0.16; 95% CI: 0.002–15.46; *p* = 0.430). Similarly, perceived stress levels did not demonstrate an independent association with high-risk group membership after accounting for other variables in the model (OR = 1.22; 95% CI: 0.82–1.83; *p* = 0.328).

The interaction between cumulative genetic risk and perceived stress levels also did not reach statistical significance (OR = 1.10; 95% CI: 0.90–1.34; *p* = 0.370), which does not allow for the assumption of a modifying effect of genetic risk on the relationship between stress and the risk of high-DEB status in the studied sample.

The model without interaction (including BMI and stress as independent predictors) previously demonstrated high explanatory power (R^2^_N_ = 0.686), and the addition of the stress × genetic risk interaction term resulted in a negligible increase in this value to 0.705 (Δ R^2^_N_ = 0.019), further indicating the absence of a meaningful improvement in model fit.

To assess the potential interaction between added sugar consumption and cumulative genetic risk with respect to the risk of DEBs, a binary logistic regression model adjusted for BMI was also constructed. In the resulting model, BMI remained a statistically significant predictor of high-risk group membership for DEBs (OR = 1.46; 95% CI: 1.06–2.03; *p* = 0.022), with each 1 kg/m^2^ increase in BMI associated with a 1.46-fold increase in the probability of being classified as a case.

Cumulative genetic risk was not associated with the likelihood of being in the high-risk group (OR = 1.43; 95% CI: 0.39–5.32; *p* = 0.591). Similarly, added sugar consumption did not demonstrate an independent association with the risk of high-DEB status after accounting for other variables in the model (OR = 1.11; 95% CI: 0.95–1.30; *p* = 0.205).

The interaction between cumulative genetic risk and added sugar consumption also did not reach statistical significance (OR = 0.99; 95% CI: 0.94–1.05; *p* = 0.832), which does not allow for the conclusion that genetic burden exerts a modifying effect on the relationship between added sugar consumption and the risk of high-DEB group membership in the studied sample.

The model without interaction (including BMI and added sugar consumption as independent predictors) previously showed R^2^_N_ = 0.453; after inclusion of the sugar × genetic risk interaction term, the value increased marginally to 0.464 (ΔR^2^_N_ = 0.011), indicating virtually no improvement in the explanatory power of the model.

Given the observed deviation from HWE for the *HTR1A* rs6295 polymorphism in the control group, a sensitivity analysis was conducted to assess whether the inclusion of this variant influenced the cumulative genetic risk score results across all regression models. The cumulative risk score was recalculated excluding rs6295, and all logistic regression analyses—both unadjusted and adjusted for BMI—were repeated. Across all models, all *p*-values for the revised genetic risk score and its interaction terms exceeded 0.1, with the sole exception of BMI, which retained its significant independent contribution (*p* = 0.014–0.015). These results are fully consistent with the primary analyses and confirm that the inclusion of the *HTR1A* variant did not materially affect the genetic risk estimates.

## 4. Discussion

The present pilot study examined the contribution of subjectively perceived stress, added sugar consumption, and genetic variants in serotonergic, neurotrophic, and dopaminergic pathways to the risk of subclinical DEBs. The study was conducted on a sample of 100 young female university students in Moscow, 40 of whom were subsequently selected from the upper and lower quintiles of the initial DEBQ distribution for case–control analysis. This demographic group is of particular interest in the context of eating disorder research, as the transition to university life coincides with a developmental period characterized by heightened vulnerability to both stress and DEBs [41,42]. Young women in this age range (median 20 years in the case group, 21 years in the control group) are exposed to multiple sources of pressure, including academic demands, social expectations, and the challenges of independent living. All of these factors may reinforce maladaptive eating behaviors and increase susceptibility to emotional eating [43].

Analysis of the DEBQ subscales revealed a consistent pattern of group differences, with the case group scoring significantly higher than the control group on all three dimensions of eating behavior (all *p* < 0.001). The most pronounced difference was observed in the emotional eating subscale, where the median score in the patient group was more than 2.5 times higher than in the control group (3.35 vs. 1.30, r = −1.0), indicating that emotional dysregulation is a key feature of the high-risk phenotype. This finding is consistent with the conceptualization of emotional eating as a maladaptive coping mechanism for negative emotions, where food serves as a readily available means of alleviating psychological distress [44]. The restrained eating subscale also demonstrated a significant difference, with median scores differing 2.3-fold between groups (3.55 vs. 1.55, r = −0.927). Food restriction in this context may reflect unsuccessful or cyclical attempts—a pattern often observed in individuals with bulimic disorders, where periods of strict dietary control alternate with episodes of binge eating [45]. Alternatively, this may indicate that the high-risk group is more consciously controlling their food intake, which may paradoxically increase vulnerability to DEBs by heightening preoccupation with food and weight. Future research should clarify whether food restriction in this population should be considered a risk factor, a consequence, or a compensatory response to episodes of binge eating. The external eating behavior subscale showed the smallest, but still significant, difference between groups (3.75 vs. 2.40, r = −0.87). Although this effect was the weakest of the three subscales, it is still noteworthy as it suggests that individuals in the high-risk group are more sensitive to external food cues—such as the sight, smell, or availability of food—and are more likely to initiate eating in response to external cues rather than internal cues of hunger or satiety.

The most compelling and consistent finding of the present study is the strong positive association between perceived stress, as measured by the PSS-14 scale, and the risk of being classified into the high-DEB group. Participants in the high-risk group (cases) demonstrated significantly higher stress scores compared to the control group (median 30.0 vs. 20.0, *p* < 0.001), and this association remained highly significant even after Bonferroni correction for multiple comparisons (adjusted α = 0.025). In binary logistic regression analyses, perceived stress emerged as the most powerful independent predictor of high-DEB status: each additional one-point increase on the PSS-14 scale corresponded to a 26% increase in the likelihood of being classified into the high-risk group in the unadjusted model (OR = 1.26; 95% CI: 1.088–1.461; *p* = 0.002) and a 44% increase after accounting for BMI (OR = 1.44; 95% CI: 1.120–1.853; *p* = 0.004). The substantial effect size (r = 0.693) and the high explanatory power of the models incorporating stress (R^2^_N_ = 0.459 in the univariate model and 0.686 after adjustment for BMI) underscore the clinical and practical significance of this association.

These results are fully consistent with the extensive literature demonstrating that chronic psychological stress potentiates emotional eating and disrupts homeostatic regulation of eating behavior through both psychological and neurobiological mechanisms [46,47,48]. At the psychological level, stress impairs cognitive control over food intake, reduces self-regulatory capacity, and promotes the use of food as a maladaptive coping strategy for negative emotions [49]. At the biological level, stress activates the HPA axis, which affects bodily systems implicated in eating behavior, particularly the balance of hunger and satiety hormones and the activity of the brain’s reward system [50,51]. Importantly, the persistence of the association between stress and high-DEB status after adjustment for BMI suggests that the effect of stress on eating behavior is not a byproduct of excess body weight (i.e., stress arising from dissatisfaction with one’s appearance) but rather represents an independent pathway linking emotional distress to maladaptive eating habits.

The relationship between added sugar consumption and high-DEB group membership yielded less robust results. In the unadjusted analysis, higher added sugar consumption was significantly associated with an increased risk of being classified as a case, with each additional gram of sugar consumed per day increasing the odds by 6.4% (OR = 1.064; 95% CI: 1.006–1.125; *p* = 0.030). This association remained significant after adjustment for BMI (OR = 1.09; 95% CI: 1.01–1.172; *p* = 0.033), indicating an independent contribution of sugar consumption not fully accounted for by body weight. However, two important considerations warrant caution in interpreting this finding: first, the effect does not survive correction for multiple comparisons (Bonferroni-adjusted α = 0.025), suggesting that the observed association may be susceptible to Type I error, particularly given the relatively small sample size and the exploratory nature of the study; second, BMI itself was a stronger and more consistent predictor of high-DEB status (OR = 1.5; 95% CI: 1.09–2.06; *p* = 0.013), and the additional contribution of sugar consumption over and above BMI appears to be limited.

These results are partially consistent with previous studies linking higher added sugar consumption and sugar-sweetened beverage intake to emotional eating and stress-induced cravings for “comfort” food [52,53]. From a mechanistic perspective, sugar consumption acutely stimulates dopaminergic reward pathways and transiently attenuates HPA-axis reactivity, providing rapid but short-lived emotional relief that may foster a state resembling addiction [54]. However, the relatively modest effect size observed in our study, combined with the attenuation of significance under more stringent statistical thresholds, raises the question of whether sugar consumption, although behaviorally relevant, may play a secondary or modulating role in the stress-eating relationship. It is also possible that the assessment of added sugar consumption using an FFQ, although widely used and validated [55,56], may be subject to inaccuracies in portion size estimation and recall bias on the part of respondents, potentially attenuating true associations. In this regard, further studies employing more objective dietary assessment methods, such as food diaries or biochemical markers of sugar intake, would be valuable.

The present study did not detect statistically significant associations between any of the four examined polymorphisms (5-HTTLPR, *5HTR1A* rs6295, *BDNF* rs6265, and *DRD2* rs1800497) and the risk of being classified into the high-DEB group. Genotype distributions between the case and control groups were largely comparable across all polymorphisms, with Fisher’s exact test yielding *p*-values ranging from 0.625 to 1.000 (Table 4), indicating that none of the investigated genetic variants individually distinguished the high-risk group from the control group. Notably, three variants were consistent with HWE, whereas rs6295 showed a significant deviation. When considered jointly, the cumulative genetic risk score, calculated as the total number of risk alleles across all four polymorphisms, also showed no significant association with high-DEB status, neither in the unadjusted logistic regression (OR = 1.269; 95% CI: 0.75–2.14; *p* = 0.369) nor after adjustment for BMI (OR = 1.18; 95% CI: 0.68–2.06; *p* = 0.555). A sensitivity analysis excluding the *HTR1A* rs6295 polymorphism from the cumulative genetic risk score did not change the overall pattern of results, as all associations remained non-significant (all *p* > 0.1) with the exception of BMI.

These null results, at first glance, appear to contradict the well-established role of serotonergic, dopaminergic, and neurotrophic pathways in appetite regulation and stress reactivity [11]; however, they are not unexpected in the context of a pilot study with a limited sample size. The absence of genetic effects may be attributable to several interrelated factors. First, the sample of 40 participants (20 per group) provides limited statistical power to detect small to moderate genetic effect sizes, which are typical for complex behavioral traits [57]. The lack of association with the cumulative risk score, despite a trend toward a positive effect (OR > 1), further suggests that the study may have been underpowered to detect subtle additive effects of multiple risk alleles. Second, subclinical DEBs represent a multidimensional phenotype that is likely influenced by a large number of genetic variants (as is the case for other behavioral traits and disorders [58,59], each with very small individual effect sizes. Examining only four polymorphisms may therefore capture only a negligible fraction of the overall heritable component. The candidate-gene strategy used here should not be interpreted as an assessment of the genetic contribution to DEBs as a whole. The four variants were selected to interrogate specific serotonergic, neurotrophic, and dopaminergic pathways. Third, population-specific genetic landscapes may differ between Russian, Asian, and Western European populations, potentially affecting the relevance of polymorphisms identified in studies on other cohorts. In this regard, we interpret the null genetic findings with caution: they do not refute the biological significance of the pathways under investigation but rather underscore the need for larger-scale studies.

To extend beyond the examination of independent genetic and environmental effects, we further investigated whether cumulative genetic risk moderated the relationship between external factors (perceived stress and added sugar consumption) and the risk of high-DEB group membership. Two separate binary logistic regression models were constructed, each including the respective environmental exposure, cumulative genetic risk score, their product term (interaction), and BMI as a covariate. In the stress-focused model, the interaction between perceived stress and cumulative genetic risk did not reach statistical significance (OR = 1.10; 95% CI: 0.90–1.34; *p* = 0.370), and the addition of the interaction term resulted in a negligible improvement in model fit (ΔR^2^_N_ = 0.019). Similarly, in the sugar-focused model, the interaction between added sugar consumption and cumulative genetic risk was not significant (OR = 0.99; 95% CI: 0.94–1.05; *p* = 0.832), with the interaction term contributing virtually no additional explanatory value (ΔR^2^_N_ = 0.011).

The obtained results suggest that, in relation to our pilot sample, the effects of perceived stress and added sugar consumption on the risk of high-DEB status appear to be independent of the investigated genetic variants, for which there are several explanations. First, as noted earlier, the limited statistical power inherent to a sample of 40 participants is problematic for detecting interaction effects. Second, although the cumulative genetic risk score offers a parsimonious approach to assessing polygenic influence, it may be too coarse to detect interaction effects, as it does not account for differential weighting of individual polymorphisms [60] nor for the possibility that different stressors may interact with distinct genetic pathways [61,62]. Third, the range of stress exposure in our sample may not have been sufficiently extreme to reveal latent genetic vulnerability. It is plausible that gene–environment interactions are only detectable when individuals are exposed to severe or chronic stress, rather than to the moderate levels typically captured by the PSS-14 in non-clinical populations [63,64]. Fourth, the timing of stress exposure may be critical: it is possible that early-life stressors, rather than current perceived stress, interact more strongly with genetic predisposition in shaping eating behavior patterns [65]. Our study did not assess adverse childhood or adolescent events; therefore, we cannot rule out the possibility that earlier environmental exposures are more relevant for the manifestation of genetic risk. Thus, further studies with larger and more diverse samples, as well as more comprehensive assessments of environmental exposures across the lifespan, are necessary to determine whether and under what conditions genetic variants interacts with psychosocial stress and dietary habits.

Several methodological strengths of the present study should be noted. First, by focusing on a homogeneous group of young female university students—a cohort particularly vulnerable to stress-induced DEBs—we reduced the influence of sociodemographic variability and were thereby able to more specifically examine the relationships between stress, dietary habits, and genetic variants in serotonergic, neurotrophic, and dopaminergic pathways. Second, the study employed a multidimensional approach, integrating validated psychometric instruments (DEBQ, PSS-14) with candidate gene analysis across four functionally significant polymorphisms. This design allowed us to simultaneously assess the contribution of environmental, behavioral, and genetic factors, as well as their potential interactions, within a unified analytical framework. Third, to the best of our knowledge, this is one of the first studies to investigate gene–environment interactions in the context of subclinical DEBs in the Russian population. The study provides preliminary data and effect size estimates that may serve as a foundation for future, larger-scale projects.

Several limitations of the study should also be noted, many of which have been discussed above. First and foremost, the small sample size (*n* = 40 in the case–control analysis) substantially limits statistical power. This limitation is particularly relevant to our null genetic findings, which cannot be interpreted as definitive evidence against the involvement of the investigated polymorphisms in predisposition to DEBs. Second, the use of self-report questionnaires (DEBQ, PSS-14, and FFQ) entails the possibility of recall bias, socially desirable responding, and inaccuracies in portion size estimation, which may attenuate or inflate the observed associations. Third, the generalizability of our findings is limited by the homogeneous nature of the sample. Fourth, within the framework of the study, only added sugar was assessed via the FFQ, and other potentially important dietary factors, such as fiber intake or consumption of ultra-processed foods, were not taken into account, despite their possible contribution to the observed outcomes [66,67]. Fifthly, although BMI was included as a covariate based on observed group differences, we acknowledge that this decision was not fully prespecified in the study protocol. However, the inclusion of BMI was methodologically justified and consistent with standard practices in the field. Future studies with larger samples and preregistered analytical plans should confirm these findings. Finally, the extreme-groups sampling strategy, while enhancing statistical efficiency for detecting between-group differences in this pilot study, inherently limits the generalizability of the findings to the general population.

## 5. Conclusions

This pilot case–control study, based on extreme-group sampling from the upper and lower quintiles of the DEBQ distribution, provides preliminary evidence that perceived psychological stress is a robust predictor of belonging to the higher-risk eating behavior group in a sample of young female university students. The association between added sugar consumption and upper-quintile DEBQ group membership, while suggestive in unadjusted analyses, did not withstand correction for multiple comparisons, precluding strong conclusions regarding its independent contribution. No significant main effects or gene–environment interactions were detected for the four candidate polymorphisms or the cumulative genetic risk score, which may reflect the limited statistical power of this pilot design rather than a genuine absence of biological effects.

Given the extreme-group sampling strategy, these findings should not be interpreted as population-level risk estimates but rather as hypothesis-generating evidence regarding the potential drivers of subclinical eating disturbances in this demographic. The results suggest that stress-reduction and emotional regulation interventions may warrant further investigation as potential approaches for preventing disordered eating behaviors in young women. However, replication in larger, population-based cohorts with longitudinal designs is essential to confirm these preliminary findings, to establish temporal relationships, and to adequately characterize the complex interplay between environmental, dietary, and genetic factors across different developmental stages and populations.

## Figures and Tables

**Table 1:** Characteristics of the participants of the first stage.

	Age, Years	BMI, kg/m^2^	Total DEBQ Scores	Emotional Eating Subscale Scores
Median	20.0	20.5	7.48	2.08
Minimum	18	16.3	4.50	0.92
Maximum	28	29.4	12.9	4.38
Me [Q1; Q3]	19.0–22.0	19.0–22.7	6.34–8.92	1.62–2.79

**Table 2:** Characteristics of the participants of the second stage.

	Controls (*n* = 20)	*p* (Controls)	Cases (*n* = 20)	*p* (Cases)
Age, years	21.0 [20.0; 21.0]	0.002	20.0 [18.8; 22.0]	0.041
BMI, kg/m^2^	19.5 [17.9; 21.3]	0.075	22.5 [20.8; 24.0]	0.237
Total DEBQ scores	5.86 [5.55; 6.23]	0.036	10.5 [10.1; 11.5]	0.139
Emotional eating *	1.30 [1.17; 1.83]	0.009	3.35 [3.08; 3.85]	0.094
External eating *	2.40 [2.27; 2.82]	0.278	3.75 [3.58; 4.00]	0.115
Restrained eating *	1.55 [1.30; 2.00]	0.002	3.55 [3.38; 4.10]	0.358
PSS-14	20.0 [17.8; 25.3]	0.472	30.0 [24.0; 33.3]	0.266
Added sugar intake	18.4 [15.1; 21.6]	0.572	35.0 [16.8; 46.0]	0.044

* Formal statistical comparisons of subscales are not performed to avoid inflating Type I error rates, as the study was not powered for subscale-level analyses.

**Table 3:** Distribution of genotypes of the entire sample at stage 2.

Polymorphism	Alleles	*n* (%)
5-HTTLPR	LL LS SS	8 (20.0) 28 (70.0) 4 (10.0)
*5HTR1A* rs6295	CC CG GG	10 (25.0) 28 (70.0) 2 (5.0)
*BDNF* rs6265	CC CT TT	25 (62.5) 14 (35.0) 1 (2.5)
*DRD2* rs1800497	GG GA AA	31 (77.5) 6 (15.0) 3 (7.5)

**Table 4:** Distribution of genotypes among case–control groups.

Polymorphism	Alleles	Controls, *n* (%)	Cases, *n* (%)	*p* ^1^
5-HTTLPR	LL LS SS ^2^	5 (25.0) 13 (65.0) 2 (10.0)	3 (15.0) 15 (75.0) 2 (10.0)	0.878
*5HTR1A* rs6295	CC CG GG ^2^	5 (25.0) 15 (75.0) 0 (0.0)	5 (25) 13 (65) 2 (10)	0.625
*BDNF* rs6265	CC CT TT ^2^	13 (65.0) 7 (35.0) 0 (0.0)	12 (60.0) 7 (35.0) 1 (5.0)	1.0
*DRD2* rs1800497	GG GA AA ^2^	15 (75.0) 4 (20.0) 1 (5.0)	16 (80.0) 2 (10.0) 2 (10.0)	0.738

^1^ Calculated using Fisher’s exact test. ^2^ Risk allele homozygotes.

## Data Availability

Data is contained within the article. Further inquiries can be directed to the corresponding authors.

## Abbreviations

The following abbreviations are used in this manuscript:

BMI	Body Mass Index
DEBs	Disordered Eating Behaviors
DEBQ	Dutch Eating Behavior Questionnaire
EDs	Eating Disorders
FFQ	Food Frequency Questionnaire
HWE	Hardy–Weinberg equilibrium
PCR	Polymerase Chain Reaction
PSS-14	Perceived Stress Scale, 14-item
VIF	Variance Inflation Factor

## References

[1] Simone M., Telke S., Anderson L.M., Eisenberg M., Neumark-Sztainer D. (2022). Ethnic/racial and gender differences in disordered eating behavior prevalence trajectories among women and men from adolescence into adulthood. Soc. Sci. Med..

[2] Keski-Rahkonen A. (2024). Eating disorders: Etiology, risk factors, and suggestions for prevention. Curr. Opin. Psychiatry.

[3] Górski M., Całyniuk B., Garbicz J., Buczkowska M., Siudmak K., Górska K., Polaniak R. (2022). Specific eating disorders–selected aspects of pathogenesis and risk factors. J. Psychiatry Clin. Psychol..

[4] Hay P. (2020). Current approach to eating disorders: A clinical update. Intern. Med. J..

[5] Nagata J.M., Garber A.K., Tabler J.L., Murray S.B., Bibbins-Domingo K. (2018). Prevalence and Correlates of Disordered Eating Behaviors Among Young Adults with Overweight or Obesity. J. Gen. Intern. Med..

[6] Foerde K., Schebendach J.E., Davis L., Daw N., Walsh B.T., Shohamy D., Steinglass J.E. (2022). Restrictive eating across a spectrum from healthy to unhealthy: Be-havioral and neural mechanisms. Psychol. Med..

[7] Ljubičić M., Matek Sarić M., Klarin I., Rumbak I., Colić Barić I., Ranilović J., Dželalija B., Sarić A., Nakić D., Djekic I. (2023). Emotions and Food Consumption: Emotional Eating Behavior in a European Population. Foods.

[8] Kerin J.L., Webb H.J., Zimmer-Gembeck M.J. (2019). Intuitive, mindful, emotional, external and regulatory eating behaviours and beliefs: An investigation of the core components. Appetite.

[9] Dakanalis A., Mentzelou M., Papadopoulou S.K., Papandreou D., Spanoudaki M., Vasios G.K., Pavlidou E., Mantzorou M., Giaginis C. (2023). The Association of Emotional Eating with Overweight/Obesity, Depression, Anxiety/Stress, and Dietary Patterns: A Review of the Current Clinical Evidence. Nutrients.

[10] Benbaibeche H., Saidi H., Bounihi A., Koceir E.A. (2023). Emotional and external eating styles associated with obesity. J. Eat. Disord..

[11] Donato K., Ceccarini M.R., Dhuli K., Bonetti G., Medori M.C., Marceddu G., Precone V., Xhufi S., Bushati M., Bozo D. (2022). Gene variants in eating disorders. Focus on anorexia nervosa, bulimia nervosa, and binge-eating disorder. J. Prev. Med. Hyg..

[12] Abou Al Hassan S., Cutinha D., Mattar L. (2021). The impact of *COMT*, *BDNF* and 5-HTT brain-genes on the development of anorexia nervosa: A systematic review. Eat. Weight. Disord..

[13] Ohrt T.K., Perez M., Liew J., Hernández J.C., Yu K.Y. (2020). The influence of temperament on stress-induced emotional eating in children. Obes. Sci. Pr..

[14] Papageorgiou L., Christou E., Louka E., Papakonstantinou E., Diakou I., Pierouli K., Dragoumani K., Bacopoulou F., Chrousos G.P., Eliopoulos E. *ADRA2B* and *HTR1A*: An Updated Study of the Biogenic Amine Receptors Reveals Novel Conserved Motifs Which Play Key Role in Mental Disorders. Advances in Experimental Medicine and Biology. Proceedings of the Worldwide Congress on “Genetics, Geriatrics and Neurodegenerative Diseases Research” (GeNeDis 2022).

[15] Bailer U.F., Kaye W.H. (2011). Serotonin: Imaging findings in eating disorders. Curr. Top. Behav. Neurosci..

[16] Manfredi L., Accoto A., Couyoumdjian A., Conversi D. (2021). A Systematic Review of Genetic Polymorphisms Associated with Binge Eating Disorder. Nutrients.

[17] Zhang Y., Wei X., Zhang W., Jin F., Cao W., Yue M., Mo S. (2024). The *BDNF* Val66Met polymorphism serves as a potential marker of body weight in patients with psychiatric disorders. AIMS Neurosci..

[18] Clarke J., Ramoz N., Fladung A.-K., Gorwood P. (2016). Higher reward value of starvation imagery in anorexia nervosa and asso-ciation with the Val66Met *BDNF* polymorphism. Transl. Psychiatry.

[19] Nonino C.B., Barato M., Ferreira F.C., Delfino H.B.P., Noronha N.Y., Nicoletti C.F., Junior W.S., Welendorf C.R., Souza D.R.S., Ferreira-Julio M.A. (2022). *DRD2* and *BDNF* polymorphisms are associated with binge eating dis-order in patients with weight regain after bariatric surgery. Eat. Weight. Disord..

[20] Olasore H.S.A., Oyedeji T.A., Faleti J.O., Ogundele O.I., Olashore A.A. (2024). Association between dopamine receptor D2 Taq IA gene polymorphism (rs1800497) and personality traits. SAGE Open Med..

[21] Aliasghari F., Nazm S.A., Yasari S., Mahdavi R., Bonyadi M. (2021). Associations of the *ANKK1* and *DRD2* gene polymorphisms with over-weight, obesity and hedonic hunger among women from the Northwest of Iran. Eat. Weight. Disord..

[22] Obregón A.M., Oyarce K., García-Robles M.A., Valladares M., Pettinelli P., Goldfield G.S. (2022). Association of the dopamine D2 receptor rs1800497 polymor-phism with food addiction, food reinforcement, and eating behavior in Chilean adults. Eat. Weight. Disord..

[23] Cardel M.I., Lemas D.J., Lee A.M., Miller D.R., Huo T., Klimentidis Y.C., Fernandez J.R. (2019). Taq1a polymorphism (rs1800497) is associated with obesity-related outcomes and dietary intake in a multi-ethnic sample of children. Pediatr. Obes..

[24] Baik J.H. (2020). Stress and the dopaminergic reward system. Exp. Mol. Med..

[25] Abizaid A. (2019). Stress and obesity: The ghrelin connection. J. Neuroendocr..

[26] Yamada C. (2021). Involvement of Ghrelin Dynamics in Stress-Induced Eating Disorder: Effects of Sex and Aging. Int. J. Mol. Sci..

[27] Hill D., Conner M., Clancy F., Moss R., Wilding S., Bristow M., O’Connor D.B. (2022). Stress and eating behaviours in healthy adults: A systematic review and meta-analysis. Health Psychol. Rev..

[28] Wu F., Vartanian L.R., Faasse K. (2025). Why Do We Eat Comfort Food? Exploring Expectations Regarding Comfort Food and Their Relationship with Comfort Eating Frequency. Nutrients.

[29] Jamar G., Ribeiro D.A., Pisani L.P. (2021). High-fat or high-sugar diets as trigger inflammation in the microbiota-gut-brain axis. Crit. Rev. Food Sci. Nutr..

[30] Lee C.-H., Giuliani F. (2019). The Role of Inflammation in Depression and Fatigue. Front. Immunol..

[31] Krupa H., Gearhardt A.N., Lewandowski A., Avena N.M. (2024). Food Addiction. Brain Sci..

[32] Wiss D.A., Avena N., Rada P. (2018). Sugar Addiction: From Evolution to Revolution. Front. Psychiatry.

[33] Sogari G., Velez-Argumedo C., Gómez M.I., Mora C. (2018). College Students and Eating Habits: A Study Using An Ecological Model for Healthy Behavior. Nutrients.

[34] Ramos-Lopez O., Panduro A., Rivera-Iñiguez I., Roman S. (2018). Dopamine D2 receptor polymorphism (C957T) is asso-ciated with sugar consumption and triglyceride levels in West Mexicans. Physiol. Behav..

[35] van Strien T., Frijters J.E.R., Bergers G.P.A., Defares P.B. (1986). The Dutch Eating Behavior Questionnaire (DEBQ) for assessment of restrained, emotional, and external eating behavior. Int. J. Eat. Disord..

[36] Barnhart W.R., Braden A.L., Price E. (2021). Emotion regulation difficulties interact with negative, not positive, emotional eating to strengthen relationships with disordered eating: An exploratory study. Appetite.

[37] Yu W., Cui L., Liu C., Wang J., Li J., Lipowski M. (2026). Social media use and disordered eating in young adults: The protective and risk pathways of physical activity and body image. Front. Psychol..

[38] Cohen S., Kamarck T., Mermelstein R. (1983). A global measure of perceived stress. J. Health Soc. Behav..

[39] Koğar E.Y., Koğar H. (2024). A systematic review and meta-analytic confirmatory factor analysis of the perceived stress scale (PSS-10 and PSS-14). Stress. Health.

[40] Boom R., Sol C.J., Salimans M.M., Jansen C.L., Wertheim-van Dillen P.M., van der Noordaa J. (1990). Rapid and simple method for purification of nucleic acids. J. Clin. Microbiol..

[41] Mallaram G.K., Sharma P., Kattula D., Singh S., Pavuluru P. (2023). Body image perception, eating disorder behavior, self-esteem and quality of life: A cross-sectional study among female medical students. J. Eat. Disord..

[42] Reichenberger J., Schnepper R., Arend A.-K., Richard A., Voderholzer U., Naab S., Blechert J. (2021). Emotional eating across different eating disorders and the role of body mass, restriction, and binge eating. Int. J. Eat. Disord..

[43] Gianini L., Foerde K., Walsh T., Riegel M., Broft A., Steinglass J.E. (2019). Negative affect, dietary restriction, and food choice in bulimia nervosa. Eat. Behav..

[44] Pengpid S., Peltzer K. (2018). Risk of disordered eating attitudes and its relation to mental health among university students in ASEAN. Eat. Weight. Disord..

[45] Pennisi F., Pinto A., Nucci D., Stacchini L., Garzitto M., Veronese N., Maggi S., Signorelli C., Baldo V., Colizzi M. (2026). Eating Disorder Risk and Its Biobehavioural Correlates in Italian University Students: The UniFood-Waste Study. Nutrients.

[46] Richardson A.S., Arsenault J.E., Cates S.C., Muth M.K. (2015). Perceived stress, unhealthy eating behaviors, and severe obesity in low-income women. Nutr. J..

[47] Sifre N., Deringer R., Prapkree L., Palacios C. (2024). Disordered Eating Attitudes and Their Association with Age, BMI, Stress, and Diet in College Students. Int. J. Env. Res. Public Health.

[48] Strete E.G., Cincu M.G., Sălcudean A. (2025). Disordered Eating Behaviors, Perceived Stress and Insomnia During Aca-demic Exams: A Study Among University Students. Medicina.

[49] Godet A., Fortier A., Bannier E., Coquery N., Val-Laillet D. (2022). Interactions between emotions and eating behaviors: Main issues, neuroimaging contributions, and innovative preventive or corrective strategies. Rev. Endocr. Metab. Disord..

[50] Castellini G., Castellani W., Lelli L., Sauro C.L., Dini C., Lazzeretti L., Bencini L., Mannucci E., Ricca V. (2014). Asso-ciation between resting energy expenditure, psychopathology and HPA-axis in eating disorders. World J. Clin. Cases.

[51] Hardaway J.A., Crowley N.A., Bulik C.M., Kash T.L. (2015). Integrated circuits and molecular components for stress and feeding: Implications for eating disorders. Genes. Brain Behav..

[52] Jacques A., Chaaya N., Beecher K., Ali S.A., Belmer A., Bartlett S. (2019). The impact of sugar consumption on stress driven, emotional and addictive behaviors. Neurosci. Biobehav. Rev..

[53] Fish-Williamson A., Hahn-Holbrook J. (2024). The Interrelationship between Stress, Sugar Consumption and Depression. Nutrients.

[54] Olszewski P.K., Wood E.L., Klockars A., Levine A.S. (2019). Excessive Consumption of Sugar: An Insatiable Drive for Reward. Curr. Nutr. Rep..

[55] Kanehara R., Goto A., Kotemori A., Mori N., Nakamura A., Sawada N., Ishihara J., Takachi R., Kawano Y., Iwasaki M. (2019). Validity and Reproducibility of a Self-Administered Food Frequency Questionnaire for the Assessment of Sugar Intake in Middle-Aged Japanese Adults. Nutrients.

[56] Buso M.E., Boshuizen H.C., Naomi N.D., Maho W., Diepeveen-de Bruin M., Balvers M.G., de Vries J.H., Harrold J.A., Halford J.C., Raben A. (2024). Relative validity of habitual sugar and low/no-calorie sweetener consumption assessed by food frequency questionnaire, multiple 24-h dietary recalls and urinary biomarkers: An observational study within the SWEET project. AM J. Clin. Nutr..

[57] Serdar C.C., Cihan M., Yücel D., Serdar M.A. (2021). Sample size, power and effect size revisited: Simplified and practical approaches in pre-clinical, clinical and laboratory studies. Biochem. Medica.

[58] Karlsson Linnér R., Biroli P., Kong E., Meddens S.F.W., Wedow R., Fontana M.A., Lebreton M., Tino S.P., Abdellaoui A., Hammerschlag A.R. (2019). Genome-wide association analyses of risk tolerance and risky behaviors in over 1 million individuals identify hundreds of loci and shared genetic influences. Nat. Genet..

[59] Grotzinger A.D., Werme J., Peyrot W.J., Frei O., de Leeuw C., Bicks L.K., Guo Q., Margolis M.P., Coombes B.J., Batzler A. (2026). Mapping the genetic landscape across 14 psychiatric disorders. Nature.

[60] Lambert S.A., Abraham G., Inouye M. (2019). Towards clinical utility of polygenic risk scores. Hum. Mol. Genet..

[61] Gan Y., Wang L., Schwarzer R., Chen G., Hu Y. (2023). Eating healthy under work stress: A gene stress interaction model. Health Psychol..

[62] Zhao M., Chen L., Yang J., Han D., Fang D., Qiu X., Yang X., Qiao Z., Ma J., Wang L. (2018). *BDNF* Val66Met polymor-phism, life stress and depression: A meta-analysis of gene-environment interaction. J. Affect. Disord..

[63] Culverhouse R.C., Saccone N.L., Horton A.C., Ma Y., Anstey K.J., Banaschewski T., Burmeister M., Cohen-Woods S., Etain B., Fisher H.L. (2018). Collaborative meta-analysis finds no evidence of a strong interaction between stress and 5-HTTLPR genotype contributing to the development of depression. Mol. Psychiatry.

[64] Delli Colli C., Borgi M., Poggini S., Chiarotti F., Cirulli F., Penninx B.W.J.H., Benedetti F., Vai B., Branchi I. (2022). Time moderates the interplay between 5-HTTLPR and stress on depression risk: Gene x environment interaction as a dynamic process. Transl. Psychiatry.

[65] Bertollo A.G., Puntel C.F., Rievers H.F.B., Brunhara E.G.S., Ignácio Z.M. (2026). Neurobiological and psychosocial mechanisms linking early life stress to the pathogenesis of eating disorders. Neuroscience.

[66] Saghafian F., Sharif N., Saneei P., Keshteli A.H., Hosseinzadeh-Attar M.J., Afshar H., Esmaillzadeh A., Adibi P. (2021). Consumption of Dietary Fiber in Relation to Psychological Disorders in Adults. Front. Psychiatry.

[67] Lane M.M., Gamage E., Travica N., Dissanayaka T., Ashtree D.N., Gauci S., Lotfaliany M., O’Neil A., Jacka F.N., Marx W. (2022). Ultra-Processed Food Consumption and Mental Health: A Systematic Review and Meta-Analysis of Observational Studies. Nutrients.

